# Is lower tourniquet pressure during total knee arthroplasty effective? A prospective randomized controlled trial

**DOI:** 10.1186/s12891-019-2636-7

**Published:** 2019-06-04

**Authors:** Tae Kyun Kim, Ankur B. Bamne, Jae Ang Sim, Ji Hyeon Park, Young Gon Na

**Affiliations:** 1 TK Orthopedic Surgery, Seongnam-si, Gyeonggi-do Republic of Korea; 2 Pioneer Hospital, New Panvel, Navi Mumbai, Maharashtra India; 3 Department of Orthopedic Surgery, Gachon University Gil Medical Center, Incheon, Republic of Korea; 4 Department of Orthopedic Surgery, CM Hospital, 13, Yeongdeungpo-ro 36-gil, Yeongdeungpo-gu, Seoul, 07301 Republic of Korea; 5 Former affiliation: Gachon University Gil Medical Center, Incheon, Republic of Korea

**Keywords:** Knee, Arthroplasty, Tourniquet, Inflation pressure, Complication

## Abstract

**Background:**

Higher tourniquet pressures may be associated with an increased risk of complications. We aimed to determine (1) whether a lower tourniquet pressure [systolic blood pressure (SBP) + 120 mmHg] is as effective as conventional tourniquet pressure (SBP + 150 mmHg) in providing a bloodless surgical field and decreasing blood loss, and (2) whether lowering the tourniquet pressure decreases tourniquet-related complications compared to conventional inflation pressure.

**Methods:**

One hundred and sixty knees in 124 patients undergoing total knee arthroplasty (TKA) were randomly allocated to either conventional (*n* = 80) or lower inflation pressure group (*n* = 80). The quality of the initial surgical field and occurrence of intraoperative blood oozing, hemoglobin drop, drained volume and calculated blood loss were assessed as efficacy variables. Safety outcome variables included post-operative pain, tourniquet site skin problems (ecchymosis, bullae, skin necrosis), and other tourniquet-related complications such as nerve palsy, venous thromboembolism, and delayed rehabilitation.

**Results:**

A comparable bloodless surgical field was successfully provided in both groups (100% vs. 99%, *p* = 1.000). One case in the conventional pressure group and two cases in the lower pressure group showed intraoperative blood oozing (*p* = 1.000), which was successfully controlled after an increase of 30 mmHg in the tourniquet inflation pressure. There was no difference in the hemoglobin drop, drained volume, and calculated blood loss. The two groups did not differ in any safety outcomes such as post-operative pain, thigh complications, and other tourniquet related complications.

**Conclusion:**

This study demonstrates that a tourniquet inflation pressure of 120 mmHg above the SBP is effective method during TKA.

**Trial registration:**

The trial was with ClinicalTrials.gov (NCT01993758) on November 25, 2013.

## Background

A tourniquet is commonly used during total knee arthroplasty (TKA) to have a clear bloodless field, which potentially reduces operative time, decreases intra-operative blood loss, and better prepares the cement-bone interface, despite the possible adverse effects associated with its use [1, 2]. Several aspects related to tourniquet use remain debatable; one among them is the pressure used to inflate the tourniquet. An optimal tourniquet pressure should be determined to balance safety and efficacy. A higher tourniquet pressure ensures the reliable function of the tourniquet; however, it may lead to a greater incidence of complications [3]. These complications are commonly related to both the duration of its use and the inflation pressure. While a lower tourniquet pressure is safer [4], it may not provide a bloodless operative field. However, no consensus exists regarding the optimal target inflation pressure among orthopedic surgeons [5].

The pressure used to inflate the tourniquet varies among surgeons. While some prefer a uniform tourniquet inflation pressure for all patients [6–8], others vary it based on the systolic blood pressure (SBP) [9–11]. The prior approach does not take the SBP into account and may result in the use of higher tourniquet pressures. On the other hand, the reported safe margin added to the SBP ranges widely, from 100 to 250 mmHg in the literature [12–15]. In our institution, adding 150 mmHg to the patient’s SBP was the routine method for determining the tourniquet inflation pressure; therefore, the mean inflation pressure was far lower than the fixed high tourniquet pressure such as 300 or 350 mmHg. Considering that higher tourniquet pressures are a risk factor for tourniquet-related complications, we sought to further reduce the tourniquet inflation pressure from our conventional method, SBP + 150 mmHg. Although there were studies which determined the tourniquet pressure as SBP + 100 mmHg [14–16], a report of 17% of failure of achieving bloodless surgical field after sharp SBP rise seemed to be not acceptable to the authors [17]. Thus, we set the target amount of reduction in the tourniquet inflation pressure as 30 mmHg rather than 50 mmHg.

We aimed to determine whether a lower tourniquet pressure [systolic blood pressure (SBP) + 120 mmHg] is as effective as conventional tourniquet pressure (SBP + 150 mmHg) in providing a bloodless surgical field and decreasing blood loss We also questioned whether lowering the tourniquet pressure can decrease tourniquet-related complications compared to conventional inflation pressure. We hypothesized that lower tourniquet pressures may achieve a bloodless surgical field similar to that achieved with conventional inflation pressure and present comparable blood loss, and that it may lead to a decrease in tourniquet-related complications.

## Methods

### Study design

This study was a single-center, double-blinded, randomized controlled trial). The sample size was calculated based on the difference in the primary outcome, namely the failure rate of the bloodless field. In previous literature, an incidence rate of 17% has been reported for the failure of achieving a bloodless field when the tourniquet pressure was determined to be 100 mmHg above the SBP [17]. We borrowed this result for the lower tourniquet pressure group (SBP + 120 mmHg) in our study, because no other data are currently available for this pressure. In addition, we experienced a 2% failure rate in the conventional pressure group (SBP + 150 mmHg) in the pilot study. Seventy-three subjects were required in each group to detect 15% of the difference in the failure rate when the power was set at 0.8, and the alpha value was set at 0.05. Accounting for a dropout rate of 10%, a total of 160 consecutive knees were enrolled in this study (80 in each group). This study was approved by the institutional review board of our institution. Written informed consent was obtained from all the participants in this study. This study was registered at www.clinicaltrials.gov **(**NCT01993758).

**Fig. 1:**
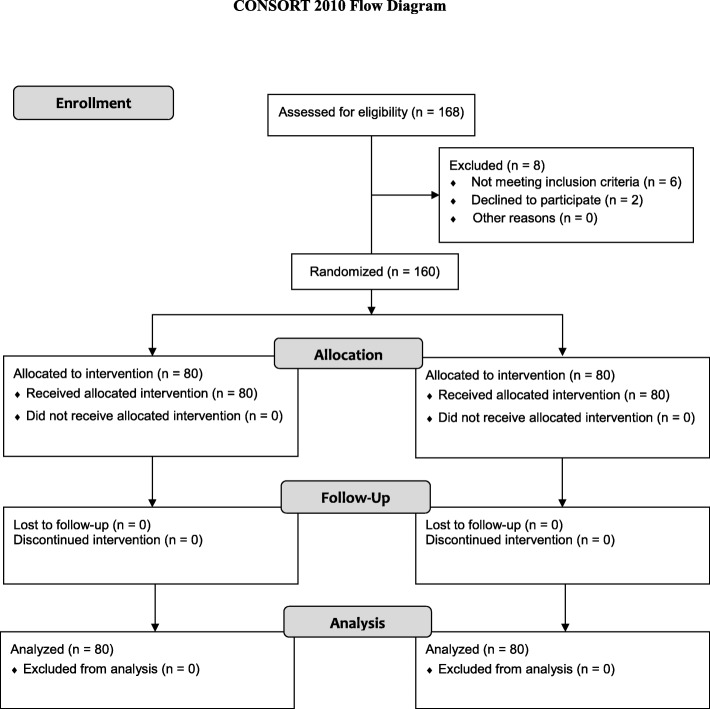
Flowchart showing enrollment of patients

Patients scheduled to undergo primary TKA (unilateral or staged bilateral TKA) for advanced knee osteoarthritis were considered eligible for inclusion. We assessed 168 knees in 130 patients undergoing unilateral or staged bilateral primary TKA between November 2013 and March 2014 for eligibility. From these patients, we excluded six patients who fulfilled at least one of the following exclusion criteria: (1) SBP measured in the ward > 200 mmHg (*n* = 0); (2) Thigh circumference > 78 cm (*n* = 0); (3) Anesthesia other than spinal anesthesia (*n* = 6); (4) peripheral vascular disease (*n* = 0); (5) refusal to participate in this study (*n* = 2) [4, 7]. The remaining 160 knees in 124 patients were randomly allocated to the conventional tourniquet pressure group (SBP + 150 mmHg, *n* = 80) or the lower tourniquet pressure group (SBP + 120 mmHg, *n* = 80) using a computer-generated randomized Table. A randomization table was created by an independent statistician, which had permuted blocks of four and six. No patients were excluded for any reason after allocation; therefore, 80 knees remained in each group in the final analysis (Fig. 1). The patients, operator and an independent investigator who collected all the information prospectively remained blind to the randomization until final data analysis. Demographic characteristics, thigh circumference, the prevalence of hypertension, diagnosis, preoperative hemoglobin, operated side, operation type, used implant, and the operation method were not different between the two groups (Table 1).

**Table 1:** Demographic features and baseline data of study groups

	Conventional [SBP + 150] (*n* = 80)	Lower Pressure [SBP + 120] (*n* = 80)	*P* value
Age (years)	71.0 (6.2)	71.8 (6.8)	0.424
Sex: female	73 (91%)	68 (85%)	0.222
Height (cm)	152.4 (6.3)	152.8 (7.8)	0.784
Weight (kg)	63.3 (9.9)	64.0 (11.1)	0.646
BMI (kg/m^2^)	27.1 (3.3)	27.4 (4.0)	0.647
Thigh circumference (cm)	47.2 (5.1)	49.2 (5.5)	0.145
Hypertension	55 (70%)	53 (66%)	0.647
Diagnosis			
Primary osteoarthritis	78 (97%)	76 (94%)	0.330
Secondary osteoarthritis	0 0%)	2 (3%)	
Inflammatory arthritis	2 (3%)	3 (3%)	
Operated side: Right	35 (44%)	42 (53%)	0.268
Operation type			
Unilateral TKA	38 (48%)	28 (35%)	0.261
Staged TKA: 1st	22 (28%)	29 (36%)	
Staged TKA: 2nd	20 (25%)	23 (29%)	
Implant			
e-motion PS Pro®	57 (71%)	64 (80%)	0.197
Genesis II®	23 (29%)	16 (20%)	
Operation method			
Conventional	63 (79%)	68 (85%)	0.305
Navigation	17 (21%)	12 (15%)	
Pre-inflation SBP (mmHg)	105.1 (15.9)	111.2 (14.8)	0.012
Initial tourniquet pressure (mmHg)	255.0 (18.1)	233.9 (15.5)	< 0.001
Tourniquet time (min)	83.1 (9.1)	82.1 (9.2)	0.486
Operative time (min)	99.3 (16.0)	99.5 (15.7)	0.925
Maximum intraoperative SBP (mmHg)	124.1 (16.8)	121.4 (15.1)	0.278
Preoperative hemoglobin (g/dL)	12.4 (1.96)	12.1 (2.1)	0.406

Data are presented as mean with the standard deviation in parentheses or number with the percentage in parentheses

*BMI* body mass index, *TKA* total knee arthroplasty, *SBP* systolic blood pressure

### Perioperative management and surgical technique

A femoral nerve block was conducted preoperatively in the operating room. A bolus dose of 30 mL of 0.375% ropivacaine was administered through an indwelling catheter, followed by a continuous infusion of 0.2% ropivacaine, and the catheter was kept remained until the third post-operative day. Spinal anesthesia was used in all the patients with 10–15 mg of 0.5% bupivacaine and 20 μg of fentanyl.

Tourniquet was applied in all patients with identical manner. A layer of elastic stockinet and three layers of cotton padding were used under the tourniquet cuff [18]. The extremity was exsanguinated with an Esmarch wrap, and the automated pneumatic tourniquet (ATS 2000, Zimmer Inc., Warsaw, IN, USA) was inflated. Based on the allocation of the subjects, the inflation pressure was set at 120 mmHg, or 150 mmHg above the last SBP measured just before tourniquet inflation (pre-inflation SBP). The surgeons were blinded to the allocation, and the inflation pressure was set by the circulating nurses who identified patient allocation from the concealed envelope. The cuff size was identical in all patients with 10 cm wide and 86 cm long.

All the operations were conducted by a single surgeon (senior author) with same manner. A midline longitudinal skin incision was used with followed medial parapatellar arthrotomy. One of the following two posterior stabilized implants were used according to the operator’s discretion: Genesis II (Smith & Nephew, Memphis, TN, USA), or e.motion PS PRO (Aesculap AG & Co. KG., Tuttlingen, Germany). Patella was resurfaced routinely in all patients. All the implants were fixated with cement (Palacos® R, Heraeus Medical, Wehrheim, Germany). After the cement was completely polymerized, the tourniquet was deflated and arterial bleeding was controlled by electrocautery. A multimodal drug cocktail were injected in the periarticular tissue for the postoperative analgesia [19]. A vacuum-assisted drain was indwelled subcutaneously. Once the hemostasis and patellar tracking was confirmed, the tourniquet was reinflated and kept inflated until the completion of the dressing [20].

All patients were given intravenous patient-controlled analgesia (PCA). All the patients underwent thrombo-prophylaxis according to our pre-determined protocol, which is an individualized-approach strategy. Patients were assessed pre-operatively for the risk of pulmonary embolism (PE) or bleeding, then were classified into four risk-stratified categories, to tailor post-operative thrombo-prophylactic therapies. The proportion of risk-stratified groups for venous thromboembolism (VTE) prophylaxis did not differ between two groups. Rivaroxaban, 10 mg, was given orally, once a day for ten days, to patients with a standard risk for both PE and bleeding and to patients with an elevated risk for PE and a standard risk for bleeding. In patients with a standard risk for PE and an elevated risk for bleeding, an intermittent pneumatic compression (IPC) device was used for seven days. In patients with an elevated risk for both PE and bleeding, an IPC device was used for seven days along with aspirin 100 mg orally once a day for six weeks. Continuous passive movement (CPM) and weight bearing with an assistive device were initiated beginning on day 2 and gradually increased over time. Patients undergoing unilateral TKA were discharged at seven days, and those who underwent staged bilateral TKA were discharged at 14 days (the 7th postoperative day after the 2nd surgery).

### Outcome measurement

The outcome measures included both intra-operative and post-operative parameters, which were evaluated within seven days after surgery. The primary efficacy outcome was the provision of a bloodless surgical field at the initiation of TKA (after skin incision, before arthrotomy). It was recorded as either present or absent. If the surgical field was obscured by blood oozing due to a sharp rise in SBP at any point after the initial exposure till the point of tourniquet deflation, it was recorded. The tourniquet pressure was increased additionally at this point by 30 mmHg at a time, for a maximum of three times. The secondary efficacy outcomes were hemoglobin drop on postoperative 2nd and 5th day, drained amount and total blood loss, which was calculated using a formula based on patient blood volume and a decrease in hemoglobin that described in several previous studies [21–23]. The secondary safety outcome measures were pain in the thigh and knee, thigh complications, and other tourniquet-related complications. The pain levels were assessed on post-operative day 2. Patients were questioned where is the most painful site among knee, thigh, or equal pain in the knee and thigh. In addition, pain level in the knee and thigh was evaluated by the patients using a visual analogue scale (VAS) from 0 to 10, with 0 indicating no pain and 10 indicating the worst pain imaginable, and a 5-point Likert scale of increasing severity (no pain to extremely severe pain). Complications at the thigh were evaluated within seven postoperative days and recorded as ecchymosis, bullae, and skin necrosis. Ecchymosis was graded as follows: Grade 0 - none; Grade 1 - present but limited to the tourniquet site, < 3 cm wide; Grade 2 - > 3 cm but still confined to the tourniquet site; Grade 3 - extending beyond the thigh. Patients were evaluated for other complications such as nerve palsy, symptomatic deep vein thrombosis (DVT), PE, and delayed rehabilitation defined as the inability to perform straight leg raise (SLR) for seven days after surgery.

### Statistical analysis

Statistical analyses were conducted with SPSS® for Windows® (version 20.0, IBM, Chicago, IL, USA) and *p* values < 0.05 were considered significant for all comparisons. The initial achievement of a bloodless surgical field and the incidence of intraoperative blood oozing owing to a sharp SBP rise, hemoglobin drop, drained volume, calculated blood loss was compared between the two groups to reveal the efficacy of the lower tourniquet inflation pressure. The postoperative knee and thigh pain, thigh complications, and other tourniquet related complications were compared between the groups to confirm the safety of each method. Statistical significance was determined using the Chi square test or Fisher’s exact test for categorical variables and the Student’s t-test for continuous variables.

## Results

The pre-inflation SBP was lower in the conventional group, but the difference was only 6.1 mmHg (105.1 vs. 111.2 mmHg, *p* = 0.012) (Table 1). The initial tourniquet inflation pressure was significantly lower in the lower tourniquet pressure group than in the conventional group (255.0 vs.233.9 mmHg; *p* <  0.001). However, the two groups did not differ with respect to tourniquet time, operative time, and the maximum intra-operative SBP.

The lower tourniquet pressure (SBP + 120 mmHg) provided a bloodless surgical field comparable to that provided using conventional tourniquet pressure (SBP + 150 mmHg), and the blood loss was similar between the two groups (Table 2). The initial quality of the bloodless surgical field was good in both groups. A bloodless surgical field was achieved in 80 knees (100%) in the conventional group and 79 cases (99%) in the lower tourniquet pressure group (*p* = 1.000). Only one patient in the lower tourniquet pressure group did not have a bloodless surgical field at the initial exposure, which was addressed by increasing the tourniquet pressure by 30 mmHg. One patient in the conventional group and two patients in the lower tourniquet pressure group had episodes of intraoperative blood oozing owing to a sharp increase in SBP, which obscured the initial bloodless surgical field. The maximum intraoperative SBP in the three patients was 136 [in the conventional group], 143 and 194 mmHg [in the lower pressure group], with the pre-inflation SBP of 100, 110, and 140 mmHg, respectively. These patients required one instance of increase in the tourniquet pressure by 30 mmHg and achieved adequate control of hemostasis. In addition, there was no difference in the hemoglobin drop on the 2nd and 5th day after surgery, drained volume and the calculated blood loss (all *p* > 0.05) (Table 1).

**Table 2:** Comparison of the efficacy of the tourniquet in both groups

	Conventional [SBP + 150] (*n* = 80)	Lower Pressure [SBP + 120] (*n* = 80)	*P* value
Bloodless surgical field (initial)	80/80 (100%)	79/80 (99%)	1.000
Intra-operative blood oozing after sharp rise in SBP	1/80 (1%)	2^*^/80 (3%)	1.000
Total failure of providing bloodless surgical field	1/80 (1%)	2^*^/80 (3%)	1.000
Hemoglobin drop on 2nd day	2.7 (1.1)	2.4 (1.3)	0.184
Hemoglobin drop on 5th day	2.8 (1.3)	2.4 (1.8)	0.076
Drained volume (ml)	23 (35)	27 (47)	0.482
Calculated blood loss (ml)	744 (256)	708 (283)	0.404

Data are presented as a number of patients with the percentage or mean values with the standard deviation in the parenthesis

^*^One patient in the lower tourniquet pressure group failed to achieve a bloodless surgical field initially and showed intraoperative blood oozing after a sharp rise in SBP

*SBP* systolic blood pressure

In terms of the safety outcomes, the two groups did not differ in any parameters, namely the pain level, thigh complications, or other tourniquet-related complications (Table 3). The VAS score and Likert scale for pain showed that there was no difference between the two groups regarding both the knee and thigh pain. The proportion of patients who suffered more severe pain in the thigh than in the knee did not differ between the groups. There was no difference in the frequency of the tourniquet-site ecchymosis between the two groups. One patient in each group developed bullae on the thigh. In addition, none of the patients developed thigh skin necrosis, nerve palsy, symptomatic DVT/PE, or delayed rehabilitation.

**Table 3:** Comparison of safety parameters among groups

	Conventional [SBP + 150] (*n* = 80)	Lower Pressure [SBP + 120] (*n* = 80)	*P* value
More painful site			
Knee	59 (74%)	60 (75%)	0.731
Thigh	15 (19%)	12 (15%)	
Knee = Thigh	6 (7%)	8 (10%)	
Knee pain (VAS)	2.8 (1.4)	3.0 (1.6)	0.451
Knee pain (Likert)			
No *or* Slight	35 (44%)	37 (46%)	0.751
≥ Moderate	45 (56%)	43 (54%)	
Thigh pain (VAS)	1.5 (1.7)	1.3 (1.6)	0.270
Thigh pain (Likert)			
No *or* Slight	61 (76%)	64 (80%)	0.566
≥ Moderate	19 (24%)	16 (20%)	
Thigh ecchymosis			
Grade 0–1	77 (96%)	73 (91%)	0.191
Grade 2–3	3 (4%)	7 (9%)	
Thigh bullae	1 (1%)	1 (1%)	1.000
Thigh necrosis	0 (0%)	0 (0%)	N/A
Nerve palsy	0 (0%)	0 (0%)	N/A
Symptomatic DVT	0 (0%)	0 (0%)	N/A
Symptomatic PE	0 (0%)	0 (0%)	N/A
Delayed rehabilitation	0 (0%)	0 (0%)	N/A

Data are presented as a number of patients and the percentage, except for the knee and thigh pain (VAS), the data for which are presented as mean and standard deviation

*VAS* visual analog scale, *DVT* deep vein thrombosis, *PE* pulmonary embolism

## Discussion

The pneumatic tourniquet is widely used during TKA despite its use being a topic of debate [24, 25]. Considering the complications associated with higher tourniquet pressures, lower inflation pressures may theoretically lead to reduced post-operative pain levels and reduced incidence of tourniquet-related complications. We undertook this study to determine if the lower tourniquet inflation pressure would be as efficacious as the conventional inflation pressures and with a lower incidence of complications. We hypothesized that a lower tourniquet pressure has comparable efficacy to conventional inflation pressure, in terms of achieving a bloodless surgical field, and that it may lead to a decrease in tourniquet-related complications.

Our findings support our hypothesis that lower tourniquet pressure would provide a bloodless surgical field comparably to conventional inflation pressure. The rationale behind inflating the tourniquet beyond the SBP allowing a certain amount of safety margin is that it accounts for the intra-operative fluctuations in blood pressure and prevents oozing in the surgical field, which might obscure the surgical field. However, using a higher inflation pressure is one of the most important reasons for complications following tourniquet use [3, 26]. Hence, use of the lowest possible inflation pressure is recommended, provided the tourniquet inflation pressure is sufficient to provide a bloodless surgical field. The mean pressure in the lower tourniquet pressure group (SBP + 120 mmHg) was 234 mmHg, which is considerably lower than the conventional group (SBP + 150 mmHg) or the fixed pressure of 300 or 350 mmHg used in other studies [6–8]. Additionally, only two patients in the lower tourniquet pressure group had an inadequate tourniquet pressure, with the incidence of tourniquet failure similar to that of a previous study [4]. This failure to achieve a bloodless surgical field was addressed by increasing the tourniquet pressure by 30 mmHg. Previous prospective randomized controlled studies that compared the lower tourniquet pressure with the higher tourniquet pressure reported that, the lower tourniquet pressure could provide a sufficient bloodless surgical field that is comparable to that provided by the higher tourniquet pressure (Table 4) [4, 12, 14–16, 27]. The mean inflation pressures in the lower inflation pressure groups were in the 223–260 mmHg range, which is similar to our result (234 mmHg), except for the mean initial inflation pressure of 182 mmHg in a study by Tuncali et al., which used arterial occlusion pressure (AOP) estimation method [27]. In addition, a previous study reported an inflation pressure of 250 mmHg as being adequate for lower extremity surgery [28], and another study found that a mean inflation pressure of 231 mmHg was adequate for the provision of a bloodless field [29]. The use of limb occlusion pressure (LOP) estimation can help individualize and decrease tourniquet inflation pressures, and modern tourniquet systems permit an automated estimation of LOP through a probe incorporated in the tourniquet system itself [4]. However, these devices may not be available at every institution, and LOP measurement involves performing an additional procedure and more time. An AOP estimation method was introduced which reported to be more simple than LOP estimation method, but this method also require measurement of extremity circumference to use estimation equation [27, 30]. Our method of adding 120 mmHg to the SBP is simple and practical in its application and has comparable results to those with conventional tourniquet inflation pressures. The efficacy of the lower tourniquet pressure, SBP + 120 mmHg, is also supported by the no difference result in the hemoglobin drop, drained volume and calculated blood loss between the two groups.

**Table 4:** Prospective randomized controlled trials reported the effect of tourniquet pressure during total knee arthroplasty

Author (Year)	Country	Group [HTP] (N) vs. [LTP] (N)	Actual tourniquet pressure (mmHg)	Duration (min)	Release timing^*^	Anesthesia	Tourniquet failure	Effect of lower tourniquet pressure
Worland (1997) [14]	USA	[350 mmHg] (28) vs. [SBP + 100 mmHg] (28)	350 vs. 230	23 vs. 22	Early	Spinal	NA	Provide bloodless operative field, Less thigh pain
Clarke (2001) [12]	England	[SBP + 250 mmHg] (10) vs. [SBP + 125 mmHg] (11)	352 vs. 223	52–94 vs. 45–91	NA	General	0/10 (HTP) 1/11 (LTP)	Less postoperative wound hypoxia
Manén Berga (2002) [16]	Spain	[400 mmHg] (41) vs. [SBP + 100 mmHg] (45)	400 vs. 260	77.4 vs. 73.7	Late	Spinal	2/45 (LTP)	Less postoperative pain, Provide bloodless operative field, Fast recovery of ROM
Ishii (2005) [15]	Japan	[350 mmHg] (30) vs. [SBP + 100 mmHg] (30)	350 vs. 238	50 vs. 48	Early	Spinal	NA	Provide bloodless operative field, No difference in perioperative blood loss
Olivecrona (2012) [4]	Sweden	[SBP + adequate margin] (76) vs. [LOP + safety margin] (83)	252 vs. 246	87 vs. 87	NA	Spinal or General	3/159 (Total)	No postoperative infections and less wound complications in cuff pressure < 225 mmHg
Tuncali (2018) [27]	Turkey	[LOP + adequate margin] (46) vs. [estimated AOP + 20mHg] (47)	200 vs. 182	70 vs. 66	NA	Combined spinal epidural	0 (Total)	Excellent or good tourniquet performance in all patients, No tourniquet related complications
The current study	Korea	HTP [SBP + 150 mmHg] (80) vs. LTP [SBP + 120 mmHg] (80)	255 vs. 234	83 vs. 82	Early (& reinflation)	Spinal	1/80 (HTP) 2/80 (LTP)	Provide bloodless operative field, No difference in postoperative pain, thigh complication, VTE, and delayed rehabilitation

^*^Timing of the tourniquet release was defined as one of two methods: late release (tourniquet was released after wound closure) or early release (tourniquet was released after implant fixation and before wound closure). In the current study, the tourniquet was released early after implant fixation for the arterial hemostasis and reinflated after hemostasis [20]

*HTP* higher tourniquet pressure, *LTP* lower tourniquet pressure, *RCT* randomized controlled trial, *SBP* systolic blood pressure, *LOP* limb occlusion pressure, *AOP* arterial occlusion pressure, *NA* not applicable, *ROM* range of motion, *HSS* Hospital for Special Surgery, *SLR* straight leg raise, *VTE* venous thromboembolism

Contrary to our expectations, using the lower tourniquet pressure did not decrease the incidence of tourniquet-related complications. However, the incidence of complications in both the study groups was rare. This may be related to the lower tourniquet pressure as well as lower ischemia time. The mean ischemia time in both our study groups was less than 100 min. A tourniquet time more than 100 min has been shown to increase complications [9]. Additionally, lower tourniquet pressures have been shown to decrease the incidence of complications following tourniquet use [4, 31]. A probable reason for the lack of difference in the incidence of complications among the two groups in our study is that our study was underpowered to detect serious complications following tourniquet use, which have a low incidence rate of one in 4200 [32]. Similarly to our findings, a study comparing lower tourniquet pressures to conventional inflation pressures (mean 252 mmHg) found no difference in post-operative pain levels [4]. However, lesser pain levels have been reported in the lower tourniquet pressure groups compared to conventional (300 or 350 mmHg) pressure groups [14, 33]. This might stem from the extent of the difference in the inflation pressures of the lower and conventional pressure groups in each study. The inflation pressure differences between the lower and conventional pressure groups in the two studies, which reported different pain levels between the groups, were 120 and 135 mmHg of mean tourniquet pressure, respectively. In the current study, the pressure difference was only 21 mmHg. One patient in each of our study groups developed skin bullae. This was probably due to frictional burns caused by an ill-fitted tourniquet. We did not experience any major complications such as thigh bullae or necrosis, nerve palsy, symptomatic DVT/PE, or delayed rehabilitation. This finding supports both methods of determining the tourniquet pressure as safely applicable in clinical practice, even when using the conventional method of 150 mmHg above the SBP.

Our study has the following several limitations: First, the majority of patients in both the groups were women. However, those selected were consecutive patients of our institute, typically showing about 90% of female predominance. This female predominance of patients undergoing TKA in the Korean population is well documented [34, 35]. Second, uniform thrombo-prophylaxis was not given to all the participants, but a risk-stratified individualized approach was used following the routine protocol of the authors’ institute. However, we think that this issue did not seriously skew the results, because all patients were randomly allocated regardless of their risk for PE and bleeding. Actually, none of the patients suffered symptomatic DVT or PE. We did not expect a considerable difference among the two groups concerning blood loss, as has been documented previously [15]. Third, the quality of the surgical field was evaluated in a subjective manner relying on the operator’s decision. However, it is difficult to rate the quality of the bloodless surgical field objectively; therefore, previous studies also employed a subjective evaluation method [4, 17, 29]. Olivecrona et al. used VAS graded by the operator after surgery regarding the quality of the bloodless surgical field and the technical difficulty caused by the quality of it [4]. Ishii et al. rated the quality of the bloodless field as poor, fair, good, or excellent, and noted any changes in the quality of the surgical field throughout the operation [17]. In the report by Reid et al., hemostasis was rated by the operating surgeon as good, adequate or failed during operation [29]. We only considered the blood oozing that definitely interfere with the surgical procedure as a failure of achieving bloodless surgical field, rather than rating into several categories or rating using visual analog scale, to minimize the effect of the subjective evaluation. In addition, there was no difference in the bleeding-related parameters, such as hemoglobin drop, drained volume, and calculated blood loss, which indirectly support the equivalent bloodless surgical field quality of the two groups. Fourth, the dosage of the spinal anesthesia was not identical to all the patients but was modified based on the body weight, which was entirely determined by the anesthesiologist. The amount of anesthetic drug may affect the pre-inflation SBP. However, there was no difference in the body weight and the BMI between the two groups, the difference of the dosage may not affect the result significantly. Fifth, there was significant difference in the pre-inflation pressure between the two groups, although the patients were randomly allocated. The difference of the pre-inflation pressure affected the difference of the initial inflation pressure, which was determined based on the pre-inflation SBP: About 21 mmHg of difference was noted, rather than mathematically anticipated 30 mmHg of difference between the two methods. Sixth, clinical significance of this study may be limited, as previous studies reported even lower tourniquet pressure method, SBP plus 100 mmHg, rather than SBP + 120 mmHg of this study [14–16]. However, considering the high failure in achieving bloodless surgical field in a study which used SBP + 100 mmHg [17], our study add an evidence of using the SBP + 120 mmHg as an alternative method.

## Conclusion

The use of lower tourniquet inflation pressure, 120 mmHg above the SBP, successfully provides a bloodless field comparable to that provided by the conventionally used higher pressure of 150 mmHg above the SBP, with a similar incidence of complications. Therefore, we recommend using a tourniquet inflation pressure of 120 mmHg above the SBP during TKA.

## Abbreviations

CPM: Continuous passive movement
DVG: Deep vein thrombosis
IPC: Intermittent pneumatic compression
LOP: Limb occlusion pressure
PCA: Patient-controlled analgesia
PE: Pulmonary embolism
SBP: Systolic blood pressure
SLR: Straight leg raise
TKA: Total knee arthroplasty
VAS: Visual analogue scale
VTE: Venous thromboembolism
